# ‘Know Your Epidemic’: Are Prisons a Potential Barrier to TB Elimination in an Australian Context?

**DOI:** 10.3390/tropicalmed3030093

**Published:** 2018-08-31

**Authors:** Nompilo Moyo, Ee Laine Tay, Justin Denholm

**Affiliations:** 1 The Victorian Tuberculosis Program at the Peter Doherty Institute, Melbourne, Victoria 3000, Australia; justin.denholm@mh.org.au; 2 Health Protection Branch, Department of Health and Human Services, Melbourne, Victoria 3000, Australia; EELaine.Tay@dhhs.vic.gov.au; 3 Department of Microbiology and Immunology, University of Melbourne, Melbourne, Victoria 3000, Australia

**Keywords:** correctional facilities, tuberculosis, treatment, incarceration

## Abstract

Globally, rates of tuberculosis (TB) cases in prisons are substantially higher than in the general population. The goal of this study was to review TB notifications in Victorian correctional facilities, and consider whether additional interventions towards TB elimination may be useful in this setting. All patients who were notified with or treated for TB in the Australian state of Victoria from 1 January 2003 to 1 December 2017 were included in this study. Descriptive analysis was performed. Demographic and treatment outcome data for individuals with and without a history of incarceration were reviewed and compared. Of the 5645 TB cases notified during the study period, 26 (0.5%) had a history of being incarcerated in correctional facilities while receiving treatment for TB. There were 73,238 inmates in Victorian correctional facilities over the same study period, meaning that approximately 0.04% of inmates were diagnosed or treated with TB disease in correctional facilities. Incarcerated individuals were more likely to have positive sputum smears and cavitation compared with nonincarcerated people with TB. There was no significant difference in treatment outcomes between the general TB population and those who had a history of incarceration during their treatment. There is a low apparent rate of TB in Victorian prisoners, and prisons do not contribute significantly to TB incidence in Victoria. Overall, TB outcomes do not differ between prisoners and nonprisoners. Ongoing efforts to sustain these lower rates and comparable outcomes in this vulnerable cohort are important for continued progress towards TB elimination.

## 1. Introduction

In many global contexts, prisons are recognised to be important sites for tuberculosis (TB) exposure, reactivation, and morbidity [1]. This phenomenon arises for a collection of reasons, including factors relating to prisoner characteristics (for example, smoking and malnutrition) and those which may arise from confinement (such as overcrowding and poor ventilation) [2,3]. Depending on health facilities available to prisoners, inadequate infection control or delays in diagnosis and treatment may further increase the risk of TB transmission and poor outcomes associated with disease [2,3].

Globally, rates of TB cases in correctional facilities are 26 times higher compared with the general population [4]. Although TB incidence rates among correctional populations in the United States have been shown to be steadily declining recently, incarcerated populations continue to experience TB at a substantially higher incidence than in the general population [5,6].

Few studies have been previously conducted on TB in Australian correctional facilities, and have primarily focused on TB infection among current prison inmates [7,8]. To our knowledge, no studies have previously considered the contribution of incarceration to overall TB incidence. Effective progress towards TB elimination requires awareness of local risk groups and consideration of appropriately targeted strategies. We sought to describe the association between TB and incarceration in Victoria, Australia, in order to evaluate the need for prison-specific approaches to TB elimination.

## 2. Materials and Methods 

Data on all cases of TB notified between 1 January 2003 and 31 December 2017 were extracted from the Department of Health and Human Services Public Health Events Surveillance System (PHESS), a centralised database containing demographic, clinical, risk factors, and contact tracing information, laboratory results, treatment details, and outcomes for all TB cases in Victoria. Medical practitioners and diagnostic laboratories are required under Victorian public health legislation to report cases of TB to health authorities [9], and all notified cases of TB are managed by the Victorian Tuberculosis Program. A microbiologically-confirmed case of TB requires culture or polymerase chain reaction (PCR) diagnosis of *Mycobacterium tuberculosis*, while clinical/radiological diagnosis may also be made by a medical practitioner experienced in TB management. Approximately 85% of TB cases in Victoria are culture-confirmed [10].

Case notes were searched for reference to incarceration, either as a clinician-identified risk factor, or through free text searches for the words ‘prison’, ‘jail’, ‘correction’, ‘custody’, ‘justice’, ‘remand’, ‘magistrate’, and for specific names of local prisons or forensic units. Case notes were reviewed and individuals with TB were eligible for inclusion in this study if they had been imprisoned in Victoria at any time, while those with a prior history of incarceration only outside of the state or overseas were excluded. Background demographic data on the general prison population was collected from the Australian Bureau of Statistics [11,12,13,14,15,16,17,18,19,20,21,22,23,24,25].

In Victoria, initial isolates of the *M. tuberculosis* complex, as well as repeat isolates from relapse cases or ‘treatment failures’, are tested for susceptibility to at least isoniazid, ethambutol, rifampicin, and pyrazinamide. Additional drugs are tested when resistance to first-line agents is found. The susceptibility tests are performed by Victorian Infectious Diseases Reference Laboratory [26].

### 2.1. Data Analysis

All data preparation and analysis was conducted using Stata version 14.0 (Stata Corp., College Station, TX, USA). Descriptive analysis was performed and comparisons in demographics, clinical characteristics, and treatment outcomes were made between cases in the general TB population and the cases that had their TB treatment in prison. Between-group comparisons were conducted with two-sided Fisher’s exact test for categorical variables due to small sample size or Wilcoxon/Mann–Whitney tests for continuous variables, with *p* values of <0.05 considered significant.

### 2.2. Ethics Statement

The data on PHESS were collected under the legislative authority of the Public Health and Wellbeing Act 2008, and therefore, approval from a Human Research Ethics Committee for this study was not required under the rules of our institutions.

## 3. Results

Of the 5645 TB cases notified between 1 January 2003 and 31 December 2017, the search strategy identified 116 potential cases for inclusion. Following case notes review, 26 cases (0.5%) met the study criteria of being incarcerated in correctional facilities at the time they were diagnosed or in receipt of treatment for TB, and were included in this analysis. With 73,238 inmates in Victorian correctional facilities over the same study period [12,13,14,15,16,17,18,19,20,21,22,23,24,25,26], this represents 0.04% (26/73,238) of inmates who were diagnosed or treated with tuberculosis disease in correctional facilities.

The characteristics of these cases and the general TB population are presented in Table 1. Prisoners were more likely to be male and were younger than the general population with TB. While site of disease did not vary significantly, prisoners were also more likely to have relapsed following previous treatment (15.4 versus 2.7%; *p* = 0.018) and to have smear-positive disease (38.5 versus 30.2%; *p* = 0.047).

**Figure 1:**
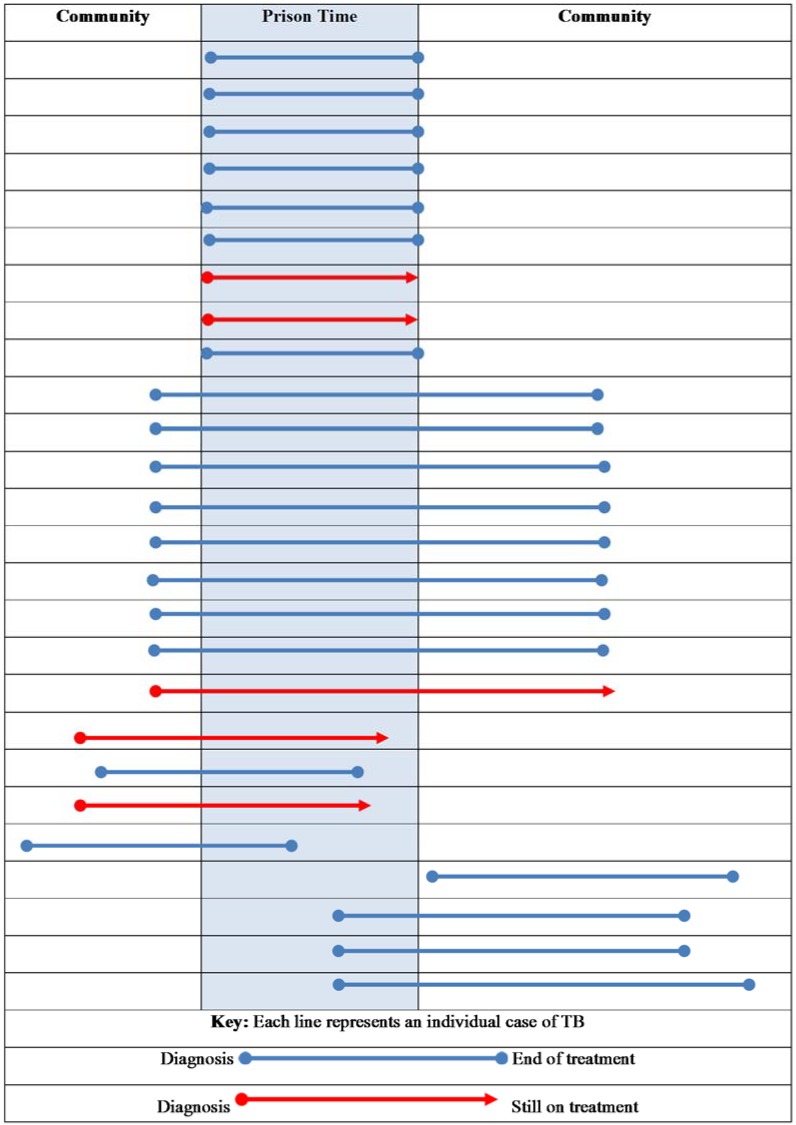
The relationship between incarceration in correctional facilities and tuberculosis diagnosis and treatment.

Figure 1 shows the relationship between the time of TB diagnosis and incarceration in correctional facilities. Seven people were diagnosed with TB while they were in prison and completed their treatment while incarcerated; one was diagnosed with TB and completed treatment shortly after release. One person was diagnosed with TB shortly after being released from prison.

Contact tracing was conducted for 13 prisoners with pulmonary TB (not shown). A total of 179 contacts was identified and screened, with 44 (24.6%) diagnosed with latent TB infection and offered preventive treatment. On six occasions, screenings were done within the prison setting. Of 59 contacts screened in the correctional facilities, two contacts had latent TB infection. Both contacts had other risk factors for TB exposure, including migration from a high TB incidence country.

**Figure 2:**
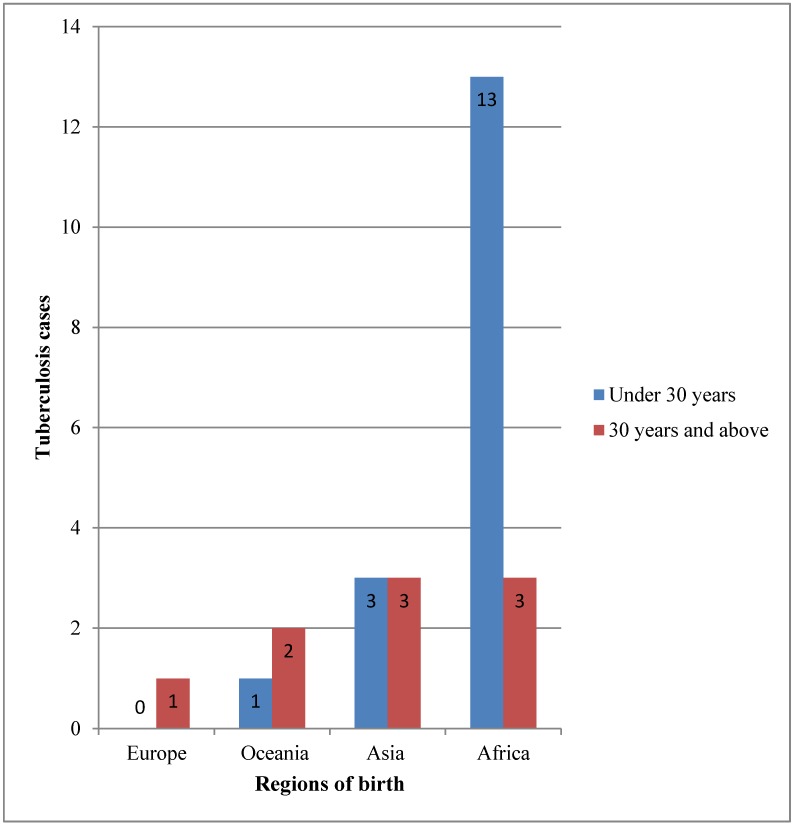
Regions of birth and ages.

Figure 2 shows the region of birth and ages of the prisoners. Ages ranged from 16 to 71 years, and 34.6% of them were aged at least 30 years. The demographic data of the general inmates in Victorian correctional facilities (not shown) and those of the inmates who were incarcerated in correctional facilities at the time they were receiving treatment for TB over the same study period were compared. There was a significant difference in age group proportions between the two groups. The majority of the inmates who were incarcerated in correctional facilities at the time they were receiving treatment for TB were aged below 30 years (65.4%), while the majority of the general inmates in Victorian correctional facilities were aged at least 30 years old (68.3%) [12,13,14,15,16,17,18,19,20,21,22,23,24,25,26]. More than half (61.5%; *p* ≤ 0.0001) of inmates who were incarcerated in correctional facilities at the time they were receiving treatment for TB were born in African countries, while in the general prison population, there were fewer inmates born in African countries (0.8%; *p* ≤ 0.0001) over the same study period [11,12,13,14,15,16,17,18,19,20,21,22,23,24,25].

The majority of individuals with TB were born in countries other than Australia, regardless of incarceration history. Of 22 prisoners with available information, 4 (18.2%) had arrived in Australia less than 5 years prior to TB diagnosis, while 13 (59.1%) arrived more than 10 years before diagnosis.

Table 2 compares the treatment outcomes between the general TB population and those who were incarcerated in correctional facilities at the time they were receiving treatment for TB. Of cases with assessable outcomes, there was no significant difference in treatment success between the general TB population (95.9% (95% CI 95.3–96.5) and those who had a history of incarceration during their treatment (91.3% (95% CI 70.4–97.9)) (*p* = 0.105). Median treatment duration did not significantly differ between prisoners and the general TB population (274 versus 214 days, respectively; *p* = 0.12). There were 52 deaths caused by TB and 64 cases lost to follow-up among the general TB population and none among those who were incarcerated in correctional facilities at the time they were receiving treatment for TB.

## 4. Discussion

Overall, we found that there is a low prevalence of TB in Victorian prisoners, and little evidence that incarceration in this context is a significant risk factor for TB disease.

While we found that treatment outcomes were similar, there are significant differences in approaches to TB management between prisoners and the general population in our setting. Most TB therapy in Victoria is provided by supported patient self-administration and involves the Victorian Tuberculosis Program nurses monitoring patients during home visits, telephone calls, and clinic attendances; directly observed therapy (DOT) is reserved for identified at-risk patients and is used in approximately 1% of cases [27]. Tuberculosis treatment in correctional facilities was provided by DOT, in keeping with medication policies for all therapy in this setting. For continuity of care, all cases that had a history of being incarcerated continued to receive DOT following release from prison. Transitions between models of care may present a range of challenges for individuals with TB, and our review found that a majority of this cohort entered and/or left prison during their period of treatment. Considered efforts are required to harmonise management and communication when transfers occur between jurisdictions or care providers, and ongoing review of policy and practice in this area will be valuable for individual patients.

Although treatment outcomes appear similar between inmates and the general population, measures of TB severity (cavitation and smear positivity) are different. This study did not explore the reasons for the differences in measures of TB severity between prisoners and the general population; therefore, further studies are recommended.

Our study was limited by its retrospective nature and relatively small number of cases within prison contexts. The method of identification of cases (keyword search in medical records) may have missed cases.

Our review focused on TB notifications in relation to prison in Victoria, and did not include testing for latent TB infection (LTBI). Data from previous Australian studies [6] has suggested that prevalence of LTBI may be higher in the prison cohort than in the general community, but has not identified attributable risk to the prison environment itself [8]. While prison may not be an independent risk factor for TB infection in our context, incarcerated individuals may still be at significant risk due to other risk factors. Prisons have been used as successful opportunities to optimise health in relation to other diseases, including hepatitis C and human immunodeficiency virus (HIV) and strategies to identify and treat LTBI in people while in prison may assist in individual health promotion.

## 5. Conclusions

There is low incidence of TB in Victorian prisoners, and this population does not contribute significantly to overall TB incidence. Incarceration does not appear to be a significant barrier to TB elimination in the Victorian setting; however, ongoing efforts to maintain TB control in at-risk populations are required. Opportunities to identify and treat LTBI in prison contexts may be valuable for future consideration and individual health promotion efforts.

## Figures and Tables

**Table 1:** Characteristics of participants.

Variables	Prison		General TB Population		*p* Value ^3^
	*n*	(%)	*n*	(%)	
Total	26		5619		
Gender					
Male	24	(92.3)	3058	(54.4)	
Female	2	(7.7)	2561	(45.6)	<0.0001
Age in years					
<30	17	(65.4)	2257	(40.2)	
≥30	9	(34.6)	3362	(59.8)	0.008
Median age (IQR)	26.5	(23–36)	33	(25–53)	0.0271 ^4^
Country of birth					
Australian	2	(7.7)	573	(10.2)	
Overseas	24	(92.3)	5041	(89.7)	
Not stated	0	(0.0)	5	(0.1)	1.000
Site of disease					
Pulmonary	13	(50)	2353	(41.9)	
Pulmonary plus other sites	5	(19.2)	759	(13.5)	
Extrapulmonary	8	(30.8)	2507	(44.6)	0.315
Sputum smear positive ^1^	10	(38.5)	941	(30.2)	0.047
Lung cavity on chest X ray (CXR) or computed tomography (CT) ^1^	7	(38.9)	6 06	(6.0)	0.099
Drug susceptibility testing ^2^					
Fully susceptible	19	(100)	3958	(91.1)	
MDR TB	0	(0.0)	80	(1.8)	
Mono- or polyresistant (not MDR TB)	0	(0.0)	290	(6.7)	
Not tested or recorded	0	(0.0)	19	(0.4)	NC
Treatment history					
New case	22	(84.6)	5377	(95.7)	
Relapse following full treatment	4	(15.4)	150	(2.7)	
Relapse following partial treatment	0	(0.0)	54	(1.0)	
Unknown/not stated	0	(0.0)	38	(0.7)	0.018

Key: Data are presented as no. (%) unless otherwise indicated. IQR: interquartile range. Multidrug resistant tuberculosis (MDR TB): defined as resistant to at least isoniazid and rifampicin. NC = not calculated. ^1^ Restricted to cases with pulmonary or pulmonary plus other sites: Prison = 18 cases; general TB population = 3112. ^2^ Culture-confirmed cases only: Prison = 19 cases; general TB population = 4347. ^3^ Derived from Fisher’s exact test unless otherwise stated. ^4^ Derived from Wilcoxon/Mann-Whitney tests.

**Table 2:** Treatment outcome.

Treatment Outcome/Assessable Outcomes	Total	TB Cases with Prison History during Treatment			General TB Population		
		*n*	%	(95% CI)	*n*	%	(95% CI)
Treatment success (completed treatment or cured)	4839	21	91.3	(70.4–97.9)	4818	95.9	(95.3–96.5)
Interrupted treatment	27	1	4.4	(0.6–26.1)	26	0.5	(0.4–0.8)
Defaulted	63	1	4.4	(0.6–26.1)	62	1.2	(1.0–1.6)
Died of tuberculosis	52	0	0.0		52	1.0	(0.8–1.4)
Failure	0	0	0.0		0	0.0	
Lost to follow-up, outcome unknown	64	0	0.0		64	1.3	(1.0–1.6)
Total assessable	5045	23	100.0		5022	100	
Non-accessible outcomes							
Transferred out of Australia	228	0	0.0		228	38.2	(34.4–42.2)
Died of other causes	178	0	0.0		178	29.8	(26.3–33.6)
Still under treatment	194	3	1.0		191	32.0	(28.4–35.9)

Definitions: Cured is defined as the bacteriologically-confirmed sputum smear and positive culture at the start of treatment and negative culture in the final month of treatment and on at least one previous occasion. Interrupted treatment is defined as treatment interrupted for two months or more but completed. Defaulted is defined as the failure to complete treatment. Failure is defined as culture-positive sputum at five months or later.
